# Covalent ISG15 conjugation to CHIP promotes its ubiquitin E3 ligase activity and inhibits lung cancer cell growth in response to type I interferon

**DOI:** 10.1038/s41419-017-0138-9

**Published:** 2018-01-24

**Authors:** Lang Yoo, A-Rum Yoon, Chae-Ok Yun, Kwang Chul Chung

**Affiliations:** 1 Department of Systems Biology, College of Life Science and Biotechnology, Yonsei University, Seoul, 03722 Korea; 2 Department of Bioengineering, College of Engineering, Hanyang University, Seoul, 04763 Korea

## Abstract

The carboxyl terminus of Hsp70-interacting protein (CHIP) acts as a ubiquitin E3 ligase and a link between the chaperones Hsp70/90 and the proteasome system, playing a vital role in maintaining protein homeostasis. CHIP regulates a number of proteins involved in a myriad of physiological and pathological processes, but the underlying mechanism of action via posttranslational modification has not been extensively explored. In this study, we investigated a novel modulatory mode of CHIP and its effect on CHIP enzymatic activity. ISG15, an ubiquitin-like modifier, is induced by type I interferon (IFN) stimulation and can be conjugated to target proteins (ISGylation). Here we demonstrated that CHIP may be a novel target of ISGylation in HEK293 cells stimulated with type I IFN. We also found that Lys143/144/145 and Lys287 residues in CHIP are important for and target residues of ISGylation. Moreover, ISGylation promotes the E3 ubiquitin ligase activity of CHIP, subsequently causing a decrease in levels of oncogenic c-Myc, one of its many ubiquitination targets, in A549 lung cancer cells and inhibiting A549 cell and tumor growth. In conclusion, the present study demonstrates that covalent ISG15 conjugation produces a novel CHIP regulatory mode that enhances the tumor-suppressive activity of CHIP, thereby contributing to the antitumor effect of type I IFN.

## Introduction

Type I interferons (IFNs) constitute a family of cytokines that are widely used in the treatment of some types of cancer and viral disease. In particular, IFN-α has a therapeutic effect in >14 types of cancer, such as melanoma, renal carcinoma, and Kaposi’s sarcoma^1,2^. IFN-α not only indirectly affects cancer by activating innate immune responses but also delays tumor cell growth by inhibiting tumor cell proliferation and angiogenesis. IFN-α upregulates the expression of numerous IFN-stimulated genes (ISGs) that directly affect tumor cell growth, apoptosis, and function of cell cycle^3^. Understanding IFN-α signaling, including ISGs, is important to clarify the mechanism of IFN-α-induced antitumor effects.

ISG15 is the first reported ubiquitin-like modifier and is highly inducible by type I IFNs^4^. Like ubiquitin, ISG15 is conjugated to specific lysine residues of target proteins (ISGylation). Similar to ubiquitination, ISGylation requires E1, E2, and E3 enzymes, all of which are induced by type I IFNs^5,6^. UbE1L and UbcH8 act as ISG15-activating (E1) and ISG15-conjugating enzymes (E2), respectively^7,8^. Three ISG15 E3 ligases—EFP, HHARI, and HERC5—have been reported^9^. Similar to reversible ubiquitination, the ISG15-deconjugating enzyme UBP43/USP18 also cleaves an isopeptide bond between ISG15 and the substrate^10^. ISGylation has been implicated in the regulation of signal transduction, ubiquitination, and antiviral responses^11–13^. ISG15 also acts as a cytokine, modulating immune responses, and as a tumor suppressor or oncogenic factor^9,14^. Proteomic studies have identified >300 cellular proteins as targets of ISGylation^15,16^; however, only some of these have been shown to be functionally regulated by ISGylation.

The carboxyl terminus of Hsp70-interacting protein (CHIP; also known as STIP1 homology and U-box containing protein 1 [STUB1]) is a chaperone-dependent E3 ubiquitin ligase. CHIP has a tetratricopeptide repeat (TPR) domain responsible for chaperone binding, a charged domain, and a U-box domain that is essential for ubiquitin ligase activity^17,18^. CHIP binds to Hsp70, Hsp90, and chaperone-bound substrates via the TPR motif and ubiquitinates substrates through the U-box domain^18,19^. Thus CHIP has dual functions as both co-chaperone and an E3 ubiquitin ligase and contributes as a regulator of a chaperone-mediated protein quality-control system^20^. In addition, CHIP has been shown to be a tumor suppressor that downregulates oncoproteins, including c-Myc, p53, HIF1-α, Smad3, and TG2, through proteasomal degradation^21–23^. Furthermore, several reports demonstrated that, depending on tumor cell context, CHIP promotes cell proliferation; this has been observed in several types of cancer^22,24^. Considering the functional diversity and physiological functions of CHIP substrates, the mechanism underlying regulation of CHIP enzymatic activity must be complex and tight to ensure normal CHIP function. According to a limited number of studies, E3 ubiquitin ligase activity of CHIP is regulated by posttranslational modifications, including phosphorylation and ubiquitination. For example, CHIP is phosphorylated by ERK5 and CDK5, enhancing its ubiquitin ligase activity^25,26^. In addition, monoubiquitination of CHIP by UBe2w is required for CHIP activation^27^.

Aside from this limited amount of data, little is known about other posttranslational modifications that may modulate CHIP activity in cells, such as via multiple ubiquitin-like modifiers. Based on the previous findings that CHIP-mediated ubiquitination and proteolysis of substrates are closely associated with type I IFN production and inflammatory signaling^28,29^, we investigated the effect of ISG15 on CHIP and its E3 ligase activity. Our results demonstrate that CHIP is modified through covalent ISG15 conjugation when cells are stimulated with IFN-α. ISGylation also enhances E3 ubiquitin ligase activity of CHIP, leading to the increase of its tumor-suppressor function against IFN stimulation.

## Results

### CHIP is a target of ISGylation

**Fig. 1:**
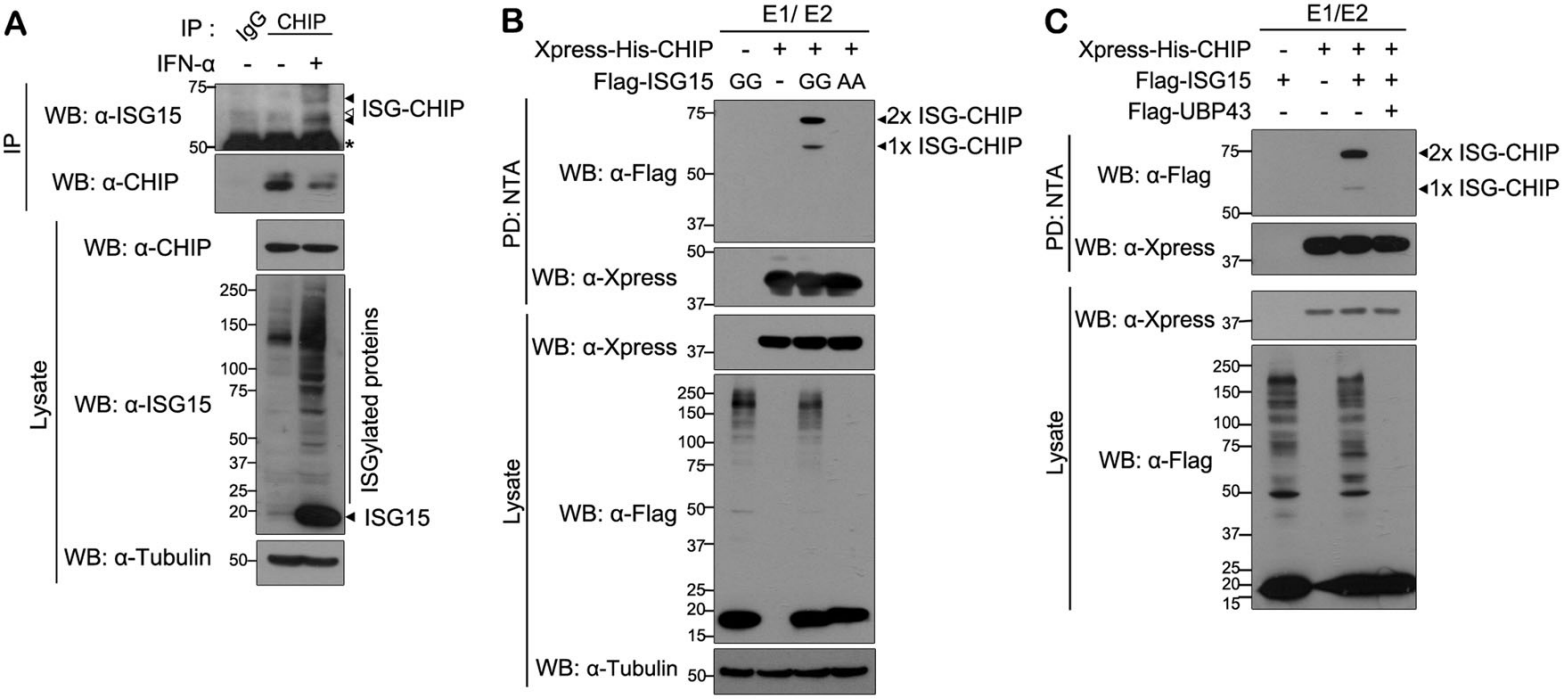
CHIP is a target of ISGylation in mammalian cells **a** A549 cells were treated for 48 h with vehicle (−) or interferon-α (1000 U/ml). Immunoprecipitation (IP) of cell lysates was carried out with anti-CHIP antibody followed by western blotting (WB) with anti-ISG15 or anti-CHIP antibody. As a negative control, cell lysates were immunoprecipitated with preimmune IgG (IgG). The presence of ISG15 and CHIP in cell extracts was determined by western blotting with the respective antibodies. Tubulin served as a loading control. Asterisk indicates IgG heavy chains. Closed and open arrowheads indicate two specific bands of ISGylated CHIP and one non-specific background band, respectively. **b** HEK293 cells were transfected for 24 h with plasmid encoding Xpress-His-CHIP, FLAG-ISG15-WT (FLAG-ISG15-GG), or its conjugation-defective mutant (FLAG-ISG15-AA). Cell lysates were subjected to NTA pull-down (PD: NTA) under denaturing conditions, followed by western blotting with anti-FLAG or anti-Xpress antibody. Expression of Xpress-CHIP and FLAG-ISG15 was verified with anti-Xpress or anti-FLAG antibody. All samples were transfected with plasmids encoding ISG15-activating enzyme UBE1L (E1) and Myc-labeled ISG15-conjugating enzyme UbcH8 (E2). Tubulin served as a protein loading control. **c** HEK293 cells were transfected for 24 h with plasmid encoding Xpress-His-CHIP, FLAG-ISG15, or FLAG-UBP43, alone or in combination. Cell lysates were subjected to NTA pull-down under denaturing conditions, followed by western blotting with the indicated antibodies. All samples were transfected with plasmids encoding UBE1L (E1) and Myc-UbcH8 (E2)

We first examined whether CHIP might be a target of covalent ISGylation in mammalian cells. When A549 cells were stimulated with IFN-α to induce ISGylation, western blot analysis demonstrated that expression of ISG15 is strongly induced, as expected, and protein ISGylation is subsequently increased (Fig. 1a). Moreover, IFN-α treatment produced two ISGylated endogenous CHIP bands (Fig. 1a). To confirm whether CHIP is covalently conjugated to ISG15, human embryonic kidney 293 (HEK293) cells were transfected with multiple components of ISG15-conjugating system. Cells were then lysed with 8 M urea-containing lysis buffer, followed by Ni-NTA pull-down analysis. Under these denaturing conditions, two ISGylated CHIP bands were clearly detected, and the pattern and sizes of the bands were identical to those detected when ISG15-GG was overexpressed but not ISG15-AA (Fig. 1b). Lastly, these two bands were undetectable when ISG15-deconjugating enzyme UBP43 was co-expressed (Fig. 1c). Taken together, these data suggest that CHIP is a target of ISGylation.

### HERC5 and EFP mediate ISGylation of CHIP

**Fig. 2:**
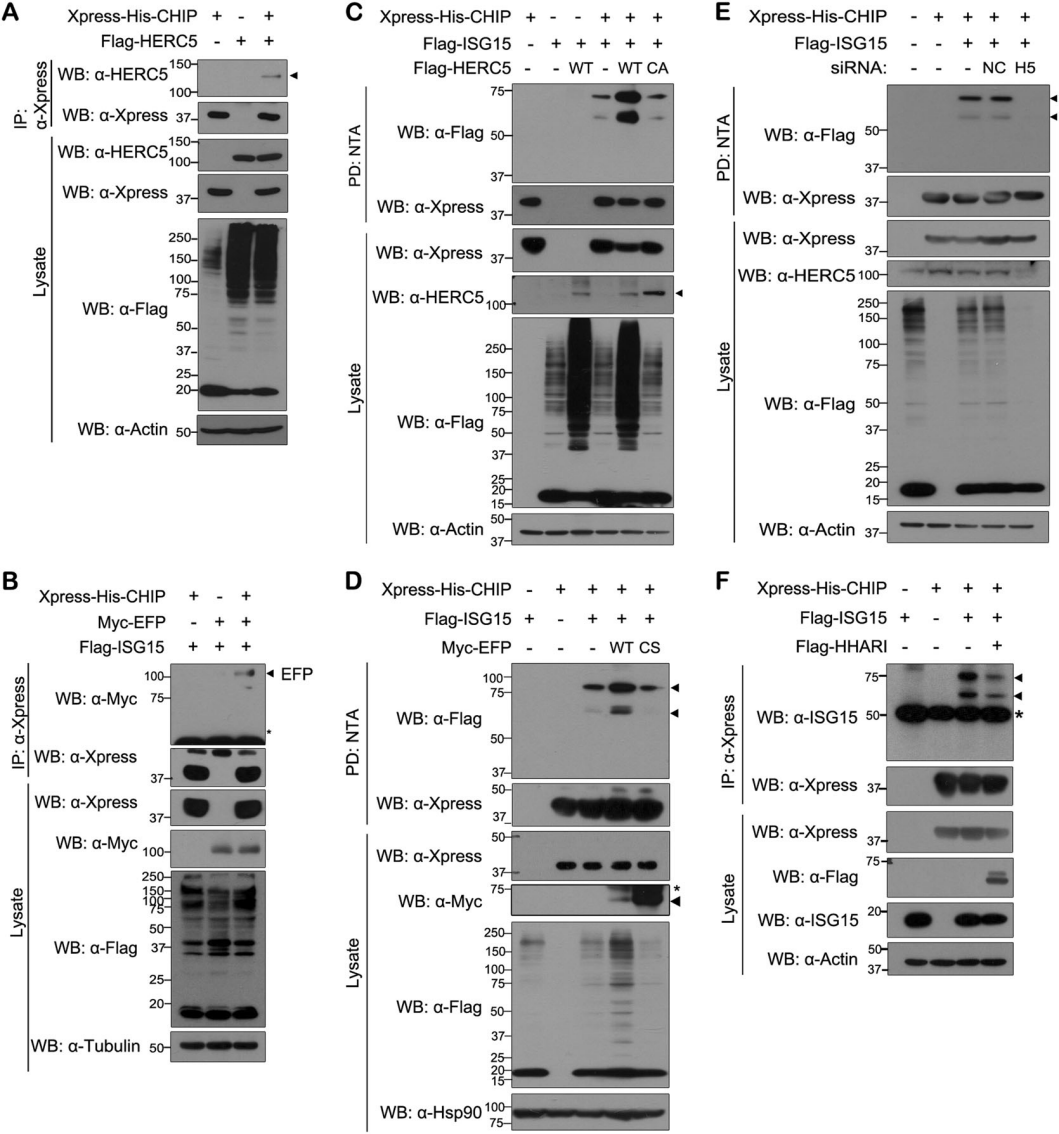
Two ISG15 E3 ligases HERC5 and EFP promote CHIP ISGylation **a**–**e** All samples were transfected with plasmids encoding UBE1L and Myc-tagged UbcH8. **a** HEK293 cells were transfected for 24 h with plasmid encoding Xpress-His-CHIP and/or FLAG-HERC5. Cell lysates were immunoprecipitated with anti-Xpress antibody, followed by immunoblotting with anti-Xpress or anti-HERC5 antibody. **b** Cells were transfected for 36 h with plasmid encoding Xpress-His-CHIP, FLAG-ISG15, or Myc-EFP, alone or in combination. Cell lysates were immunoprecipitated with anti-Xpress antibody, followed by western blotting with anti-Myc or anti-Xpress antibody. **c** HEK293 cells were transfected for 24 h with plasmid encoding Xpress-His-CHIP, FLAG-ISG15, FLAG-HERC5-WT, or its catalytically inactive mutant FLAG-HERC5-CA, alone or in combination. Cell lysates were subjected to NTA pull-down (PD: NTA) under denaturing conditions followed by western blotting with anti-FLAG or anti-Xpress antibody. **d** Cells were transfected for 24 h with plasmid encoding Xpress-His-CHIP, FLAG-ISG15, Myc-EFP-WT, or Myc-tagged catalytically inactive EFP mutant Myc-EFP-CS, alone or in combination. Cell lysates were subjected to NTA pull-down, as in **c**. **e** HEK293 cells were transfected for 48 h with nonspecific control scrambled siRNA (NC), *HERC5*-siRNA (H5), or plasmid encoding Xpress-His-CHIP or FLAG-ISG15, alone or in combination. Cell lysates were subjected to NTA pull-down as in **b**. **f** Cells were transfected for 36 h with plasmid encoding Xpress-His-CHIP, FLAG-ISG15, or Flag-HHARI, alone or in combination. Cell lysates were immunoprecipitated with anti-Xpress antibody, followed by western blotting with anti-Flag or anti-Xpress antibody. Asterisk indicates IgG heavy chains, and closed arrowhead indicates predicted or right size band

To date, three E3 ISG15 ligases have been reported^9^—EFP, HHARI, and HERC5. Next, we examined whether CHIP binds to HERC5 and/or EFP. Co-immunoprecipitation experiment revealed that CHIP binds to both HERC5 and EFP (Fig. 2a, b). In addition, wild-type HERC5 or EFP clearly increased CHIP ISGylation (Fig. 2c, d). However, this effect was not seen with their catalytically inactive mutants (Fig. 2c, d). Similarly, knockdown of *HERC5* suppressed CHIP ISGylation (Fig. 2e). These results suggest that both ISG15 E3 ligases, HERC5 and EFP, can mediate CHIP ISGylation.

### Lys143, Lys144, Lys145, and Lys287 are the major ISGylation sites in CHIP

**Fig. 3:**
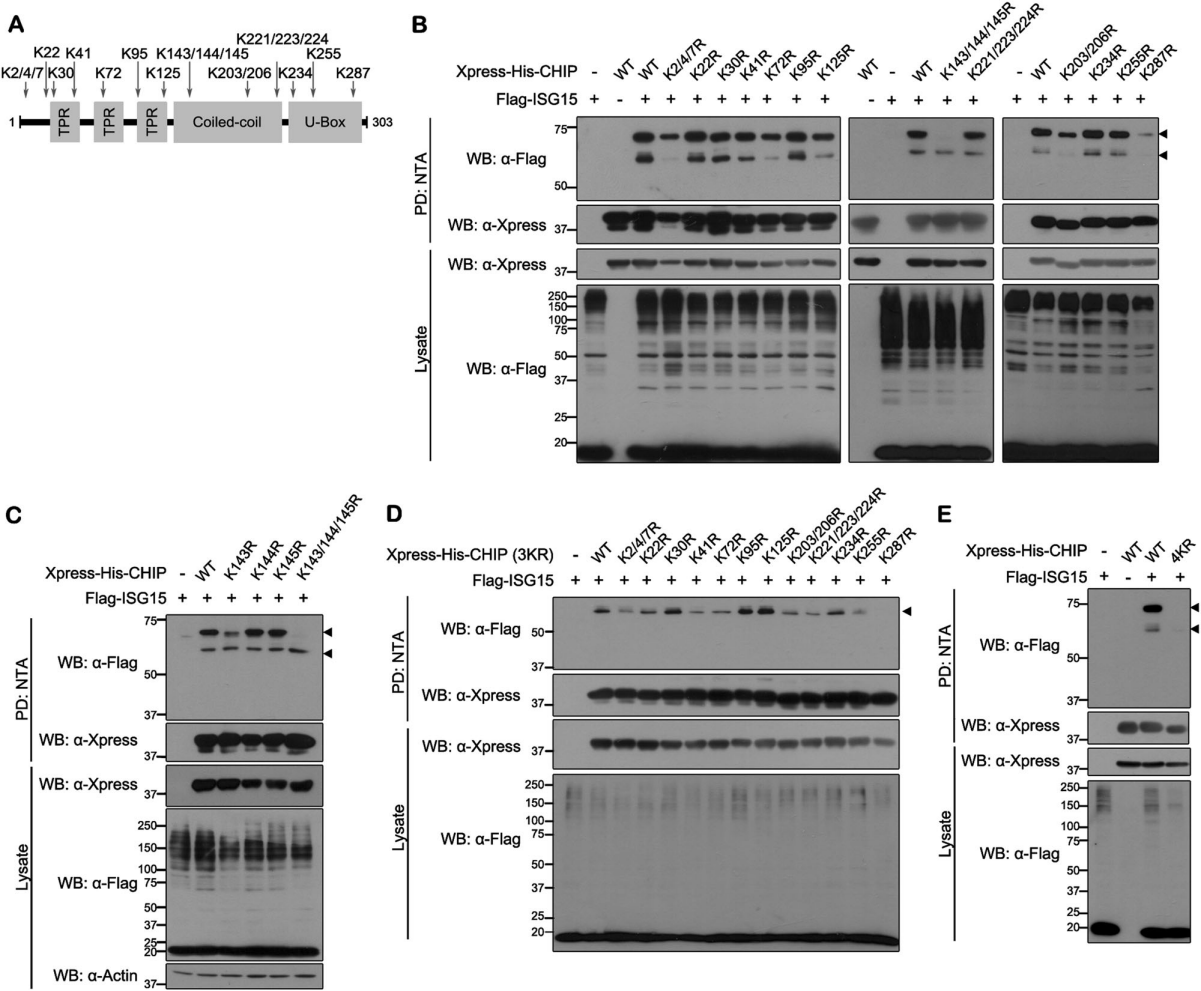
Identification of covalent ISG15 conjugation sites in CHIP **a** Schematic diagram of all lysine residues in full-length CHIP. **b**–**e** HEK293 cells were transfected for 24 h with plasmid encoding Xpress-His-CHIP (WT), the indicated point mutants, or FLAG-ISG15, alone or in combination. Cell lysates were subjected to NTA pull-down (PD: NTA), followed by western blotting with anti-FLAG or anti-Xpress antibody. In the CHIP-3KR mutant, the three indicated lysine residues have been substituted with arginines (K143/144/145 R). The CHIP-4KR mutant has the same point mutations, plus substitution of K287 with arginine (K143/144/145/287 R). All samples in **b**–**e** were transfected with plasmids encoding UBE1L and Myc-UbcH8. Closed arrowhead indicates predicted or right size band

It was previously reported that ISG15 is conjugated to lysine residues on target proteins as a monomer, not as a polymer or after polymerization^30^. As two bands of CHIP are detected under conditions of ISGylation, we hypothesized that at least two CHIP Lys residues are ISGylated. To determine the ISGylation target residues in CHIP, we constructed several mutants, each with a point mutation resulting in substitution of 1 of the 20 Lys with Arg (Fig. 3a). Because it was highly possible that two or three consecutive or neighboring Lys residues might be employed as alternative ISGylation sites, four additional mutants having double or triple mutations were generated in those cases (Fig. 3a).

Screening of all 13 mutants revealed that there was no change in CHIP ISGylation with the mutations K22R, K30R, K41R, K95R, K221/223/224R, K234R, or K255R (Fig. 3b). Whereas the lower CHIP band, representing the conjugation of a single ISG15 moiety, was effectively abolished with mutations K2/4/7R, K72R, K125R, and K203/206R, the upper band still remained to a greater or lesser extent. In addition, the formation of the upper ISGylated CHIP band was completely abolished with the mutations K143/144/145R, and the intensity of band was dramatically diminished with the CHIP-K287R mutant (Fig. 3b). These two mutants also displayed greatly reduced intensity of the lower CHIP-ISG15 band, suggesting that ISG15 might be conjugated to two of these four lysine residues. To identify two residues as ISG15-conjugation sites, we next generated three CHIP mutants having a single mutation at K143R, K144R, or K145R and examined whether they were ISGylated. As shown in Fig. 3c, K144R and K145R mutants were still efficiently ISGylated, but K143R was ISGylated much less (Fig. 3c). These data indicate that a single one of these three residues does not serve as the targeting residue.

To identify another ISGylation site in CHIP, we generated 12 additional mutants having four point mutations of CHIP in which the remaining Lys residue of CHIP-K143/144/145R (CHIP-3KR) was replaced by Arg, respectively. As shown in Fig. 3d, e, among these quadruple mutations of CHIP, the mutation at K143/144/145/287R (CHIP-4KR) completely abolished ISGylation. We could expect that two ISG15 conjugations of CHIP occurred sequentially. Both of the two bands for ISGylation of CHIP-K287R were significantly reduced, but the upper band for ISGylation of K143/144/145R mutant was mainly affected. Based on these results, we speculated that ISG15 is primarily conjugated at K287 in CHIP and then secondly ISGylated at K143/144/145 in CHIP.

Taken together, our data suggest that CHIP has two ISGylation sites. Interestingly, the conjugation of monomeric ISG15 to CHIP at K143, K144, or K145 appears to always occur and possibly randomly.

### ISGylation stimulates the E3 ubiquitin ligase activity of CHIP

We next investigated how ISGylation affects the biochemical and functional properties of CHIP. Previous reports demonstrated that several stimuli, including lipopolysaccharide treatment, alter the intracellular localization of CHIP, promoting its nuclear translocation from the cytoplasm in many cases^31,32^. Based on these reports, we first evaluated whether ISGylation affects the intracellular localization of CHIP (Figure S1A). ISG15 was predominantly expressed in the cytoplasm, and ISGylated CHIP was also detected in cytoplasm. However, we could not observe the increase of ISGylated CHIP level in the nucleus. Moreover, the localization pattern of conjugation-defective mutant CHIP-4KR was nearly identical to that of wild-type CHIP. These results indicated that CHIP ISGylation occurs in cytoplasm, and this modification does not cause the nuclear translocation of CHIP. (Figure S1A). As ISGylation also regulates the steady-state level of its target proteins^12,33^, we next assessed the stability of CHIP-WT and CHIP-4KR. Measurement of CHIP half-life using cycloheximide revealed that CHIP-WT was slowly degraded over 24 h and that CHIP-4KR had a similar turnover rate (Figure S1B). Taken together, these results imply that ISGylation does not affect the subcellular localization or stability of CHIP.

**Fig. 4:**
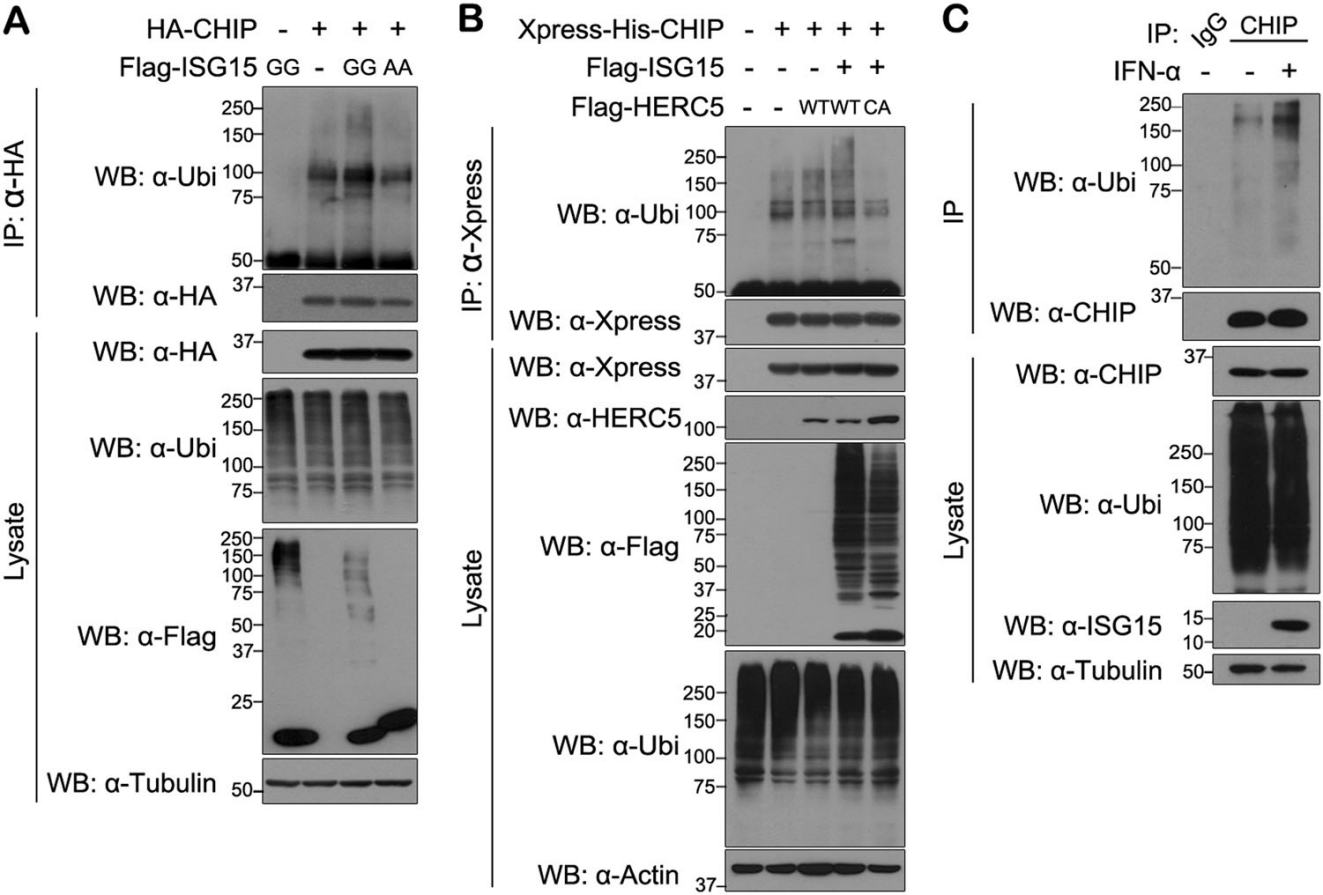
ISGylation increases CHIP autoubiquitination **a**, **b** All samples were transfected with plasmids encoding UBE1L and Myc-UbcH8. **a** HEK293 cells were transfected for 24 h with plasmid encoding HA-CHIP, FLAG-ISG15-GG, or FLAG-ISG15-AA, alone or in combination. Cells were treated for an additional 6 h with 10 μM MG132. Cell lysates were immunoprecipitated with anti-HA antibody, followed by western blotting with anti-ubiquitin antibody. Tubulin served as a loading control. **b** HEK293 cells were transfected for 24 h with plasmid encoding Xpress-His-CHIP, FLAG-ISG15, FLAG-HERC5-WT, or FLAG-HERC5-CA, alone or in combination, and treated for an additional 6 h with 10 μM MG132. Cell lysates were immunoprecipitated with anti-Xpress antibody, followed by western blotting with anti-ubiquitin antibody. Actin served a as loading control. **c** A549 cells were treated for 48 h with vehicle (−) or IFN-α (1000 U/ml). Immunoprecipitation of cell lysates was performed with preimmune IgG or anti-CHIP antibody, followed by western blotting with anti-ubiquitin or anti-CHIP antibody. Tubulin served as a loading control

We next examined whether ISGylation affects the ubiquitin E3 ligase activity of CHIP. Like many other ubiquitin E3 ligases, the activity of ISGylated CHIP was assessed based on its capacity for autoubiquitination. As shown in Fig. 4a, autoubiquitination of CHIP was slightly increased by ISG15-GG, but not by ISG15-AA. In addition, autoubiquitination of CHIP was further increased by HERC5-WT, but not by HERC5-CA (Fig. 4b). Likewise, IFN-α treatment, which was shown to promote CHIP ISGylation, also enhanced autoubiquitination of endogenous CHIP in A549 cells (Fig. 4c). However, as ISGylation does not affect CHIP stability (Figure S1B), the increase in CHIP autoubiquitination may not cause its degradation possibly through proteasomal machinery.

**Fig. 5:**
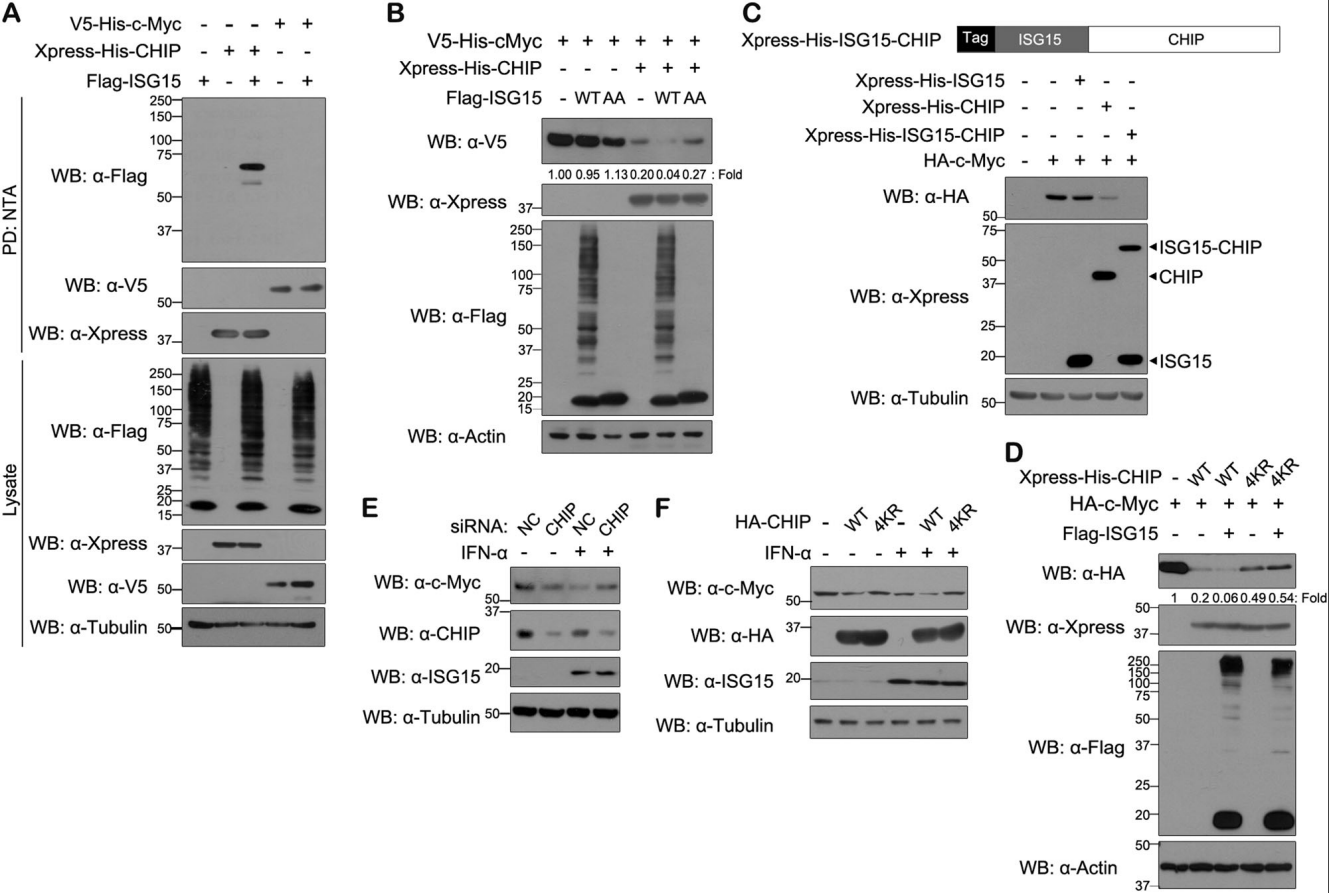
IFN-α treatment reduces c-Myc level via CHIP ISGylation **a**, **b** All samples were transfected with plasmids encoding UBE1L and Myc-UbcH8. **a** HEK293 cells were transfected for 24 h with plasmid encoding Xpress-His-CHIP, V5-His-c-Myc, or FLAG-ISG15-GG, alone or in combination. Cell lysates were subjected to NTA pull-down, followed by western blotting with anti-FLAG, anti-V5, or anti-Xpress antibody. Tubulin served as a loading control. **b** HEK293 cells were transfected for 24 h with plasmid encoding Xpress-His-CHIP, V5-His-c-Myc, FLAG-ISG15-WT, or FLAG-ISG15-AA, alone or in combination. Cell lysates were western blotted with the indicated antibodies. Values below top panel indicate the intensity of c-Myc bands relative to that of loading control α-actin. **c** (Upper) Schematic representation of recombinant Xpress-ISG15-CHIP fusion protein. (Lower) HEK293 cells were transfected for 24 h with plasmid encoding Xpress-His-ISG15, Xpress-His-CHIP, Xpress-His-ISG15-CHIP, or HA-c-Myc, alone or in combination. Cell lysates were western blotted with the indicated antibodies. Tubulin served as loading control. **d** HEK293 cells were transfected for 24 h with plasmid encoding Xpress-His-CHIP-WT, Xpress-His-CHIP-4KR, HA-c-Myc, or FLAG-ISG15, alone or in combination. All samples were transfected with plasmids encoding UBE1L and Myc-UbcH8. Cell lysates were western blotted with the indicated antibodies. Values below top panel indicate the intensity of c-Myc bands relative to that of loading control α-actin. **e**, **f** A549 cells were transfected for 24 h with nonspecific control siRNA (NC, **e**), *CHIP*-siRNA (CHIP, **e**), or plasmid encoding HA-CHIP-WT or HA-CHIP-4KR **f**, alone or in combination. Cells were treated for an additional 36 h with vehicle (−) or IFN-α (2000 U/ml). Cell lysate were then western blotted with the indicated antibodies

Previous reports revealed that CHIP binds to and negatively regulates the steady-state levels of many proteins, such as c-Myc and TG2, through its ubiquitin E3 ligase activity^21,23^. To confirm the effect of ISGylation on CHIP ligase activity, we measured the level of c-Myc, which should not be a direct target of ISGylation, before and after CHIP ISGylation. We confirmed that c-Myc is not a substrate for ISG15 modification (Fig. 5a). Moreover, western blotting analysis revealed that the presence of CHIP-WT alone greatly reduced the level of c-Myc (by 80%), as expected. Consistent with the results of CHIP autoubiquitination assays (Fig. 4), cells expressing CHIP-WT displayed a further decrease in c-Myc level by >95%, in the presence of ISG15-GG, but not with ISG15-AA (Fig. 5b). Since the c-Myc level was reduced to ~4% in cells overexpressing CHIP-WT plus wild-type ISG15-GG, the activity of ISGylated CHIP toward c-Myc degradation was enhanced by ~1.2-fold higher than CHIP-WT. As a control, c-Myc levels in the cells expressing either ISG15-GG or ISG-AA alone were similar. These results suggest that ISGylation stimulates the ubiquitin E3 ligase activity of CHIP.

These findings were further supported by the assay using the artificial ISG15 conjugation-mimetic mutant of CHIP. Several studies have demonstrated that ISG15 fusion to a target protein can mimic the ISG15-modified state^30,34^. Based on these reports, we similarly produced a construct (ISG15-CHIP) in which ISG15 is fused to the N-terminus of CHIP. Interestingly, this mimetic form of ISGylated CHIP significantly reduced c-Myc level (Fig. 5c). On the other hand, CHIP-4KR mutant used as a negative control showed much lower c-Myc degradation activity. Furthermore, co-expression of CHIP-4KR with ISG15 did not enhance CHIP-4KR activity and c-Myc proteolysis, as expected (Fig. 5d). All of these results indicate that ISGylated CHIP possesses enhanced activity in negative regulation of its substrate c-Myc.

Lastly, we examined the effect of CHIP ISGylation on c-Myc degradation in response to IFN-α stimulation. IFNs were shown to suppress c-Myc mRNA and protein expression in cancer cells^35^. In addition, IFN-β caused a reduction in the steady-state level of c-Myc protein by increasing degradation through the 26S proteasome^36^. Consistent with these reports^37^, IFN-α treatment of A549 cells resulted in a remarkable decrease in intracellular c-Myc level (Fig. 5e). Further, c-Myc was restored to control levels by *CHIP* knockdown (Fig. 5e). Moreover, overexpression of CHIP-WT accelerated c-Myc degradation in IFN-α-treated cells, whereas CHIP-4KR overexpression did not (Fig. 5f).

Taken together, these data demonstrate that ubiquitin E3 ligase CHIP plays an important role in IFN-α-induced c-Myc degradation through CHIP ISGylation.

### IFN-α-induced CHIP ISGylation inhibits cell growth via c-Myc degradation

**Fig. 6:**
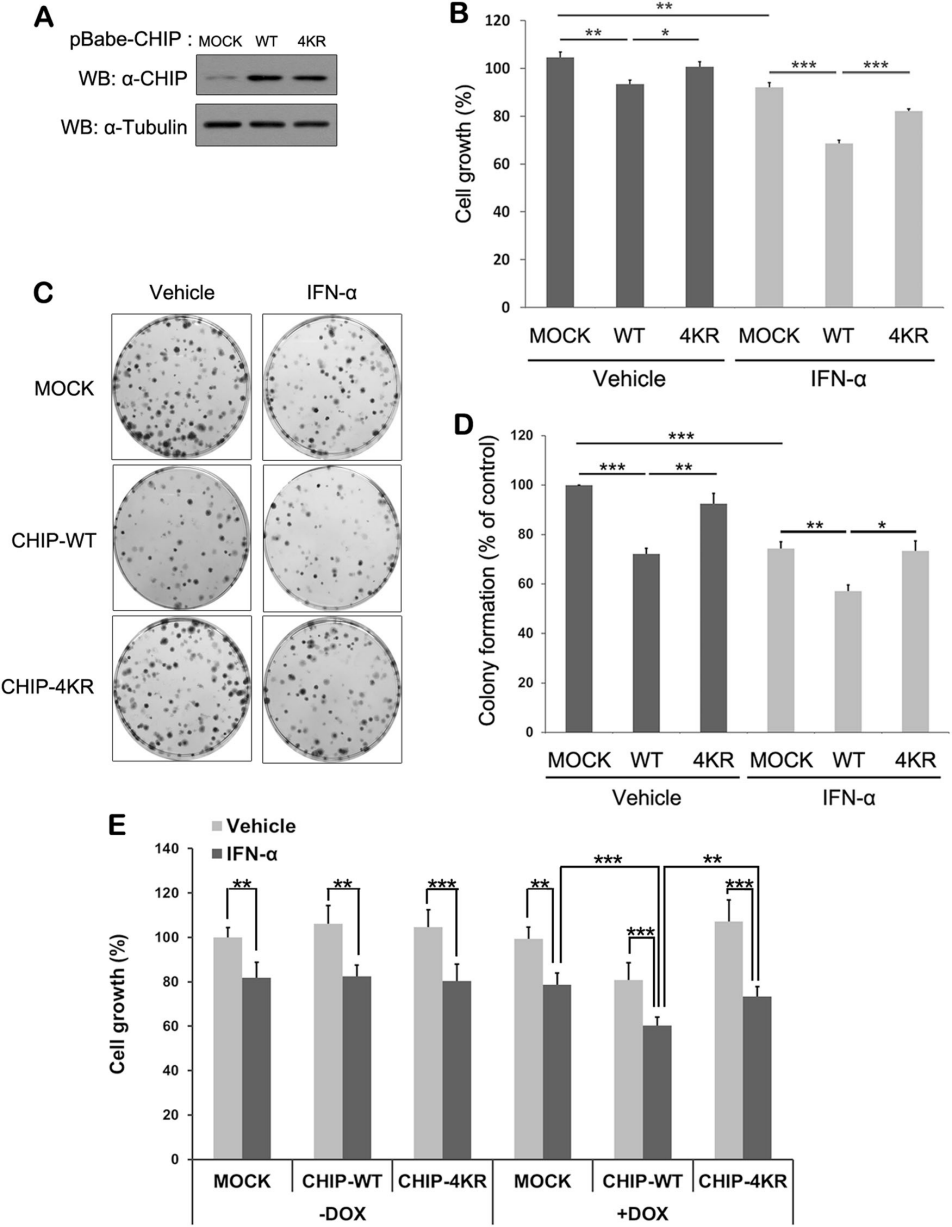
IFN-α-induced CHIP ISGylation causes inhibition of A549 cell growth and colony formation **a** Cell lysates were prepared from A549 cells stably expressing mock vector (Mock), CHIP-WT, or CHIP-4KR followed by western blotting with anti-CHIP antibody. **b** A549 cell lines stably expressing mock vector, CHIP-WT, or CHIP-4KR were treated for 48 h with vehicle or IFN-α (4000 U/ml) and then counted using the CCK-8 assay. Data are represented as the mean ± standard error of the mean (S.E.M.) of three independent experiments (*n* = 9; *P < 0.05; **P < 0.01; ***P < 0.001). **c**, **d** A549 cell lines stably expressing mock vector, CHIP-WT, or CHIP-4KR were cultured for 10 days, treated for an additional 7 days with vehicle or IFN-α (2000 U/ml), and stained with crystal violet **c**, and percentage of colony formation (mean ± S.E.M) was assessed in three independent experiments (*P < 0.05; **P < 0.01; ***P < 0.001) **d**. **e** A549 cells were infected with the lentivirus carrying the MOCK, CHIP-WT, or CHIP-4KR and cultured in the presence or absence of doxycycline for 5 days. Cells were additionally treated for 48 h with vehicle or IFN-α (4000 U/ml) and then counted using the CCK-8 assays. Data are represented as the mean ± standard error of the mean (S.E.M.) of three independent experiments (*n* = 9; *P < 0.05; **P < 0.01; ***P < 0.001)

Type I IFNs delay the progression of various cancer cells by inhibiting cell proliferation and promoting apoptosis^37,38^. IFNs suppress expression of *c-Myc* mRNA and protein before causing growth arrest^39^, which underlies its antitumor activity. Based on these findings, we next investigated the effect of CHIP ISGylation on growth of lung cancer cells. To do this, we established lines of A549 human epithelial lung carcinoma cells stably expressing CHIP-WT or CHIP-4KR (Fig. 6a). Consistent with the previous report, IFN-α treatment greatly attenuates A549 cell proliferation. Moreover, overexpression of CHIP-WT further promotes the inhibitory effect of IFN-α on cell growth, which was not seen with CHIP-4KR (Fig. 6b). This inhibitory action of IFN-α on A549 cell growth was further confirmed by colony-formation assay (Fig. 6c). Cells treated with IFN-α produced colonies that were slightly smaller in size and number than those of control mock-transfected cells (Fig. 6c). Furthermore, overexpression of CHIP-WT facilitated reduced colony formation after IFN-α treatment, whereas CHIP-4KR restored the colony-formation pattern seen in control cells.

To verify the inhibitory effect of CHIP and its ISGylation on A549 cell growth, we compared growth rate in A549 cells conditionally expressing CHIP-WT or CHIP-4KR through the Tet-On system. As expected, there was no change of cell proliferation in all groups under no treatment of doxycycline, which confirmed the validity of Tet-on system. Moreover, IFN-α treatment in the absence of doxycycline caused a remarkable reduction of cell proliferation in all samples. Compared with cells transduced with mock vector under doxycycline treatment, there was a further decrease of growth in the mock-transduced cells treated with IFN-α alone or expressing doxycycline-induced CHIP-WT. Furthermore, IFN-α treatment plus CHIP-WT induction inhibited the growth of cells in an additive manner. In contrast, CHIP-4KR-expressing cells exhibited much less inhibition of cell growth induced by IFN-α treatment (Fig. 6e). These results were identical to those from A549 cells stably expressing mock, CHIP-WT, or CHIP-4KR.

Taken together, these results indicate that IFN-α-induced ISGylation of CHIP negatively regulates the growth and colony formation of A549 human lung cancer cells.

### The tumor-suppressive effect of CHIP is enhanced through ISGylation

**Fig. 7:**
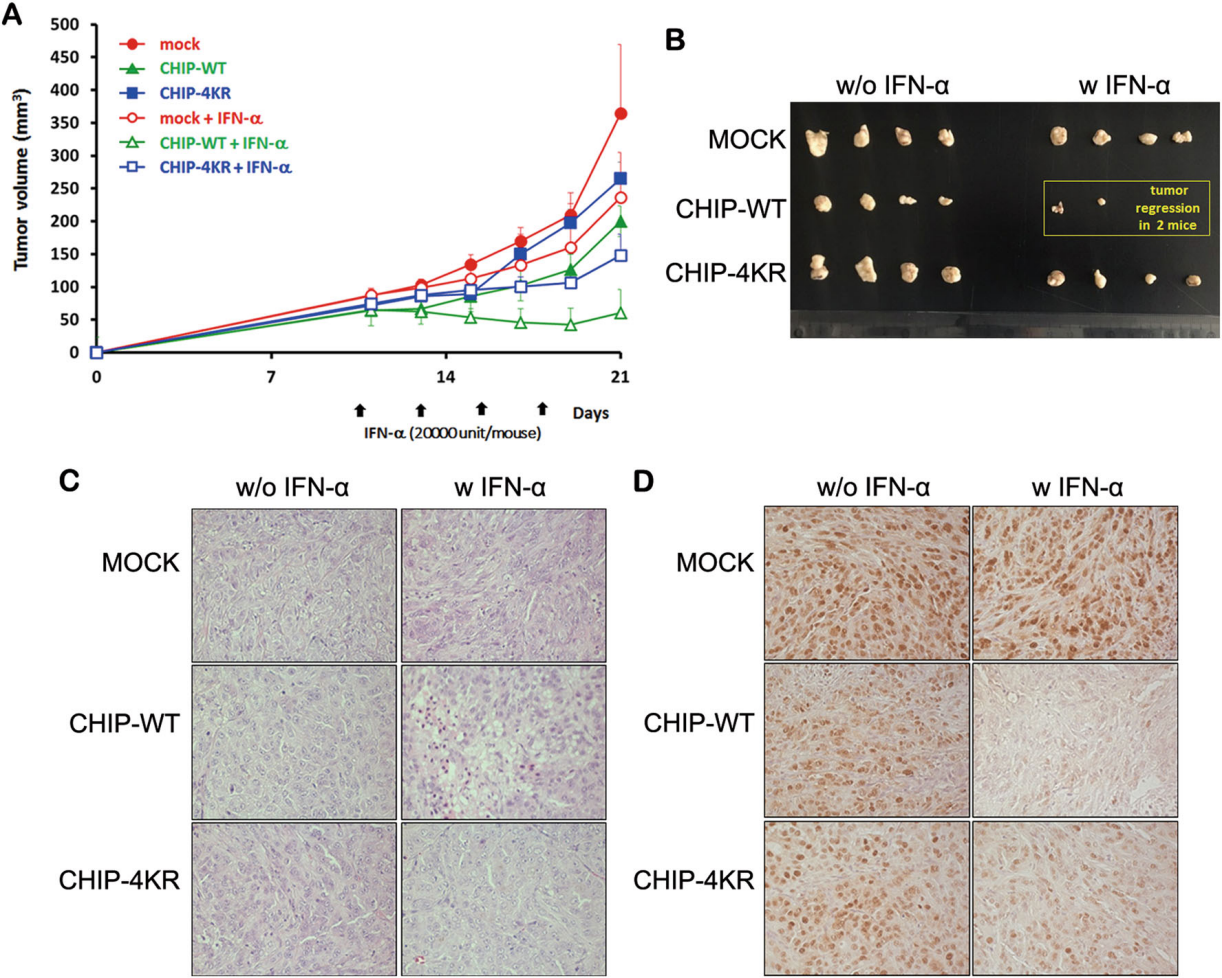
Tumor growth inhibition induced by CHIP expression **a** Mice bearing CHIP variants (i.e., Mock, CHIP-WT, or CHIP-4KR) expressing A549 tumor were given intratumoral injections of IFN-α on days 11, 14, 17, and 20. Tumor growth was monitored every 3 days until the end of the study. Values represent the mean ± S.E.M. for four animals per group. **b** Tumor tissues harvested from each group were visualized. **c**, **d** Tumors were harvested on day 21 for histological analysis. H&E staining **c** and immunohistochemical staining of PCNA **d** were performed on tumor sections from each group of mice

Next we assessed the tumor-suppressive effect of CHIP ISGylation using different A549 stable cell lines (mock, CHIP-WT, or CHIP-4KR). As shown in Fig. 7a, A549 tumors expressing mock, CHIP-WT, or CHIP-KR grew rapidly in the absence of IFN-α treatment with average tumor size reaching 364.9 ± 104.5, 200.4 ± 60.3, or 264.8 ± 25.4 mm^3^ by day 21 following cell implantation, respectively. In marked contrast, A549 tumors expressing CHIP-WT or CHIP-4KR treated with IFN-α reached an average size of 61.0 ± 35.5 or 147.8 ± 32.5 mm^3^, showing a 69.5 or 44.2% growth inhibition, respectively, when compared with A549 tumors expressing CHIP-WT or CHIP-4KR without IFN-α. Interestingly, CHIP-WT-expressing A549 tumors in combination with IFN-α led to complete tumor regression in two out of the four mice, whereas no complete regression was observed in the CHIP-4KR plus IFN-α group (Fig. 7b). Throughout the course of the study, no systemic toxicity, such as diarrhea, weight loss, or cachexia, was observed.

Taken together, these results suggest that CHIP expression inhibits A549 tumor growth, and this effect is further potentiated by IFN-α-induced CHIP ISGylation.

### CHIP-mediated necrosis and decreased proliferation tumor cell is augmented by ISG15 conjugation

To further characterize the antitumor effect bestowed by CHIP and its ISGylation, tumor tissues were examined by histological and immunohistochemical analysis. Hematoxylin and eosin (H&E) staining revealed that most of the tumor masses were necrotic in A549-CHIP-WT treated with IFN-α, whereas necrotic lesions in A549-mock or A549-CHIP-4KR were only detectable in limited tumor regions (Fig. 7c). Moreover, CHIP-WT-expressing tumor tissues treated with IFN-α exhibited much lower level of proliferating cell nuclear antigen (PCNA; estimated approximately 10-fold lower) compared with CHIP-WT-expressing tumors without IFN-α, implying that IFN-α-mediated CHIP ISGylation and its activation can efficiently suppress tumor cell proliferation and promote necrosis of lung tumors (Fig. 7d).

## Discussion

IFNs modulate the function of the immune system in various ways, consequently affecting cell proliferation, differentiation, and apoptosis. Specifically, type I IFNs (IFN-α/-β) initiate a signaling cascade through the Janus-activated kinase–signal transducer and activator of transcription factor pathway and the regulation of IFN regulatory factors (IRFs). These events finally lead to the transcription of about 500 ISGs^38,40^. Of the hundreds of ISGs, in the present study we focused on ISG15, which modulates the function of target proteins by ISGylation^9^. ISG15 and ISGylated proteins usually act as tumor suppressors in several types of human cancer cell lines, including breast, lung, blood, and cervical cancer cells, and myelogenous leukemia cells^41–43^. Therefore, identification of novel targets of ISGylation may be important to elucidate the mechanism underlying the tumor-suppressive action of type I IFNs. Here we identified CHIP as a novel ISGylated protein. Several reports have shown that CHIP acts as a negative regulator of IFN production through degradation of IRF-1 and RIG-I^28,29^. In addition, the present findings suggest that stimulation of CHIP activity after ISGylation inversely and negatively alters IFN production.

In regard to the effect of CHIP on cancer cell growth, CHIP primarily impedes tumor progression through degradation of several cell-proliferation-, cell-survival-, and angiogenesis-related proteins, such as ErBb2, c-Myc, EGFR, and HIF-1α, in diverse cancer types (i.e., breast, gastric, and pancreatic cancer). On the contrary, CHIP was also strongly expressed in different types of cancer, including gallbladder carcinoma, esophageal squamous cell carcinoma, and glioma. Furthermore, CHIP promotes the degradation of several tumor-suppressor genes, including FoxO1, p53, PTEN, and IRF-1, suggesting that CHIP also functions as an oncogene. The overall results indicate that CHIP has opposing roles in different cancers, either promoting or suppressing tumor progression, depending on the cellular environment^22^.

Consistent with the previous report^22^, the present study also supports the tumor-suppressive action of CHIP in breast cancer cells, which is mediated mainly through downregulation of c-Myc and its oncogenic potential. Similar to the previous report^36^, c-Myc level was decreased in IFN-α-stimulated cells, and CHIP ISGylation accelerated c-Myc degradation. Moreover, IFN-α treatment after CHIP overexpression leads to inhibition of A549 cell growth, which can be rescued upon overexpression of CHIP-4KR. These results suggest that the antitumor effect of type I IFN is largely dependent upon and mediated through CHIP ISGylation.

Notwithstanding the diverse functional roles and physiological importance of CHIP, the molecular mechanisms underlying modulation of its activity have not been extensively explored. To date, CHIP activity appears to be regulated primarily by interactions between proteins^44^. Concerning posttranslational modifications, ubiquitin E3 ligase activity of CHIP is regulated through phosphorylation and proteasome-independent ubiquitination. For instance, monoubiquitination of CHIP at Lys2 is mediated by E2 enzyme Ube2W, which recruits deubiquitinating enzyme ataxin-3, blocks CHIP activity, and efficiently terminates CHIP-mediated proteolysis^27^. In addition, CDK5 phosphorylates CHIP at Ser20 and negatively regulates its activity by disrupting the interaction between CHIP and its substrate^25^. ERK5 and LIMK have also been reported to be CHIP-interacting kinases; however, their effect on CHIP activity has not been clarified^44^. The present study revealed that CHIP may be a target of covalent ISG15 conjugation, which is similar to ubiquitination and stimulates CHIP ubiquitination activity. Based on the structural similarities between multiple ubiquitin-like moieties, our results strongly indicate that CHIP may also be modified by other UBL targets, such as SUMO, FAT10, and NEDD8, which is an interesting hypothesis for further testing. The presence of multiple regulatory and/or backup modes for CHIP activity retrospectively confirms its functional and physiological importance and demonstrates that tight regulation of CHIP is important for cell physiology.

Many ISG15-conjugated proteins control cellular responses to viral infection, IFN, and DNA damage. For example, PCNA, p53, ΔNp63α, and parkin are targets of ISG15 conjugation under genotoxic stress^45,46^. The current work identifies an additional target of ISGylation. Our study also reveals for the first time that ISGylation affects protein quality control during the IFN-triggering inflammatory process.

Here we identified two ISGylation sites within CHIP, Lys287, and, independently, Lys143, Lys144, or Lys145. The Lys143/144/145 residues are located in the central coiled-coil region, whereas the Lys287 is present in the C-terminal U-box domain of CHIP. The coiled-coil region is essential for CHIP dimerization, which is a prerequisite for its catalytic E3 ligase activity, and the U-box domain is also important for activity through interacting with the E2 enzyme^44^. To further identify the mechanism underlying enhanced CHIP E3 ubiquitin ligase activity after ISGylation, we examined functional differences between wild-type CHIP and its conjugation-defective CHIP-4KR mutant, focusing on the aspect of homodimerization and/or affinity of binding to substrates, E2 enzyme, or Hsp70/90 (data not shown). Extensive evaluation of these parameters revealed no noteworthy differences. Based on these data, we presume that ISGylation may induce a subtle change in CHIP structure, possibly altering the conformation of the active site, and consequently stimulating the catalytic ubiquitin-transfer reaction. Although we present no evidence to support this hypothesis, advanced structural analysis using high-resolution biophysical tools would be expected to demonstrate such a subtle change in CHIP conformation after ISGylation in the future.

In conclusion, we propose a novel mechanism of tumor-suppressive effect of IFN-α via CHIP ISGylation in A549 human lung cancer cells.

## Materials and methods

### Animal studies

Six-week-old male athymic nude mice were purchased from Charles River Korea (Seongnam-si, Gyeonggi Province, Korea) and maintained in a laminar air flow cabinet under specific pathogen-free environment. All facilities were approved by Association for Assessment and Accreditation of Laboratory Animal Care. All animal studies were performed according to the institutionally approved protocols of Hanyang University.

### Materials

Dulbecco’s modified Eagle’s medium (DMEM), fetal bovine serum (FBS), and Lipofectamine 2000 were purchased from Invitrogen (Carlsbad, CA, USA). Monoclonal anti-FLAG antibody was purchased from Sigma-Aldrich (St. Louis, MO, USA). Anti-c-Myc, anti-CHIP, anti-ubiquitin, anti-HA, anti-actin, and anti-tubulin antibodies were purchased from Santa Cruz Biotechnology (Santa Cruz, CA, USA). Polyclonal anti-HERC5 antibodies were purchased from Novus Biologicals (Littleton, CO, USA). Anti-ISG15 antibody was purchased from Boston Biochem (Cambridge, MA, USA). Anti-histone H3 antibody was purchased from Abcam (Cambridge, UK). Peroxidase-conjugated anti-rabbit and anti-mouse secondary IgGs were purchased from Millipore (Billerica, MA, USA). MG132 was purchased from A. G. Scientific (San Diego, CA, USA). Protein A-Sepharose and Ni-NTA agarose beads were purchased from GE Healthcare Life Sciences (Piscataway, NJ, USA) and Invitrogen, respectively. Enhanced chemiluminescence (ECL) reagent was purchased from AbClon (Seoul, Korea). IFN-α was purchased from PBL Assay Science (Piscataway, NJ, USA).

### DNA constructs and RNA interference

The mammalian construct encoding human wild-type CHIP N-terminally tagged with Xpress-6x-His (pcDNA4-Xpress-6x-His-CHIP) was kindly provided by D.H. Lee (Seoul Women’s University, Seoul, Korea). Plasmid encoding HA-tagged wild-type CHIP (pRK5-HA-CHIP), retroviral vectors encoding human wild-type CHIP (pBABE-puro-CHIP-WT), and lentiviral vectors encoding human wild-type CHIP (pLVCT-CHIP-WT-tTRKRAB) were produced by PCR amplification of pcDNA4-Xpress-CHIP as template using Prime STAR-HS DNA Polymerase (TAKARA, Shiga, Japan) and subcloned into vectors pRK5-HA and pBABE-puro and pLVCT-tTRKRAB, respectively. Plasmids encoding FLAG-tagged wild-type ISG15 (FLAG-ISG15-WT), its conjugation-defective mutant (pFLAG-ISG15-AA), wild-type UBE1L (pcDNA-UBE1L), Myc-tagged UbcH8, and Myc-tagged EFP were all kindly provided by C.H. Chung (Seoul National University, Seoul, Korea). Plasmid encoding FLAG-tagged HERC5 was provided by K. Hochrainer (Weill Cornell Medical College, New York City, NY, USA). Plasmids pFLAG-HHARI and pLVCT-tTR-KRAB were gifts from D.E. Zhang (University of California at San Diego, San Diego, CA, USA) and P. Aebischer and D. Trono (Swiss Federal Institute of Technology, Lausanne, Switzerland), respectively. Mammalian constructs encoding HA- and V5-tagged wild-type c-Myc were provided by H.D. Youn (Seoul National University) and I.K. Chung (Yonsei University, Seoul, Korea), respectively. To make constructs encoding various point mutants of CHIP, site-directed mutagenesis was performed using the QuikChange XL Site-directed Mutagenesis Kit (Agilent Technologies, Santa Clara, CA, USA). All cDNA sequences were verified by sequencing (COSMO Genetech, Seoul, Korea). The siRNA targeting human *CHIP* (siRNA no. 1146289) and control-scrambled siRNA (catalog no. SN-1013) were purchased from Bioneer (Daejeon, Korea).

### Cell culture and DNA transfection

HEK293 and human lung carcinoma A549 cells were maintained in DMEM containing 10% FBS and 100 U/ml penicillin-streptomycin. Cells were grown at 37 °C in 5% CO_2_. All DNA transfections were performed using Lipofectamine 2000, according to the manufacturer’s protocol.

### Co-immunoprecipitation assay and western blotting analysis

For detection of protein ISGylation, cells were lysed in RIPA buffer (50 mM Tris, pH 7.4, 150 mM NaCl, 1% Triton X-100, 0.5% sodium deoxycholate, 0.1% SDS, and protease inhibitor cocktail containing 0.2 mM phenylmethylsulfonyl fluoride, 1 µg/ml aprotinin, 1 µg/ml leupeptin, 1 mM Na_3_VO_4_, and 10 mM NaF). For immunoprecipitation, cell lysates including 0.5 mg protein in cell lysis buffer were incubated with 1 µg of appropriate antibody overnight at 4 °C and then with 30 µl of an equal volume of Protein A-Sepharose bead suspension for 2 h at 4 °C with gentle rotation. Beads were pelleted by centrifugation and washed five times with lysis buffer (50 mM Tris, pH 7.5, containing 1% Nonidet P40, 150 mM NaCl, 10% glycerol, and protease inhibitor cocktail). Immunocomplexes were dissociated by boiling in 2× sodium dodecyl sulfate-polyacrylamide gel electrophoresis (SDS-PAGE) sample buffer, separated by SDS-PAGE, and transferred to a nitrocellulose membrane. Membranes were then blocked in Tris-buffered saline with Tween (20 mM Tris, pH 7.5, 137 mM NaCl, and 0.1% Tween 20; TBST) containing 5% non-fat dry milk for 1 h at room temperature and incubated overnight at 4 °C in TBST containing 3% non-fat dry milk and the appropriate primary antibody. Membranes were washed three times in TBST, incubated with secondary horseradish-peroxidase-coupled IgG for 2 h at room temperature, and signal was visualized using ECL reagent.

### Ni-NTA pull-down analysis

After DNA transfection for 24 h, cells were lysed with urea-containing lysis buffer (0.1 M NaH_2_PO_4_/Na_2_HPO_4_, pH 7.4, 8 M urea, 10 mM imidazole, and protease inhibitor cocktail). After incubation of lysates with Ni-NTA agarose, precipitates were washed three times with the same buffer containing 50 mM imidazole and then eluted with the same buffer containing 500 mM imidazole. The samples were resuspended in SDS sample buffer for 5 min at room temperature, followed by separation by SDS-PAGE.

### Preparation of cytosolic and nuclear fractions

Cells were scraped in ice-cold phosphate-buffered saline and resuspended in hypotonic buffer (10 mM HEPES, pH 7.9, 1.5 mM MgCl_2_, 10 mM KCl, 0.5 mM dithiothreitol [DTT], and protease inhibitor cocktail). The cells were incubated for 10 min on ice and then lysed in 0.5% NP-40, followed by vortex mixing for 5 s. Cell lysates were centrifuged at 13,000 × *g* for 5 min at 4 °C. The supernatants served as cytosolic fractions. The nuclear pellet was washed with hypotonic buffer and resuspended in hypertonic buffer (27 mM HEPES, pH 7.9, 2 mM MgCl_2_, 560 mM NaCl, 270 mM EDTA, 33% glycerol, 0.5 M DTT, and protease inhibitor cocktail). The pellets were lysed in 1% NP-40 and incubated for 20 min on ice, followed by centrifugation at 13,000 × *g* for 20 min at 4 °C. The supernatants served as nuclear fractions.

### Generation of A549 cells stably expressing wild-type CHIP or its ISGylation-defective mutant

A549 cell lines stably overexpressing wild-type CHIP or its ISGylation-defective mutant were generated by retroviral transduction. Retroviral particles were generated by cotransfection of HEK293T cells for 48 h with two plasmids (Gag-pol and VSV-G) together with retroviral vector pBabe as control (A549-Control), pBabe-CHIP-WT (A549-CHIP-WT), or pBabe-CHIP-4KR (A549-CHIP-4KR) using Lipofectamine PLUS. Medium containing viral particles was collected and filtered through a 0.45 μm filter. A549 cells were infected with each retrovirus and selected with puromycin (5 μg/ml; Sigma-Aldrich).

### Inducible expression of CHIP using Tet-on system in A549 cells

HEK293T cells were cotransfected with one of doxycycline-inducible Tet-on plasmids (pLVCT-tTRKRAB, pLVCT-CHIP-WT-tTRKRAB, or pLVCT-CHIP-4KR-tTRKRAB) plus two plasmids (Gag-pol and VSV-G) for generation of lentiviral particles. Media containing viral particles from each sample were collected and filtered through a 0.45 μm filter. For transduction, media containing each lentivirus were added to A549 cells. Following 16 h of incubation, cells were washed with DMEM media and split, and doxycycline at a final concentration of 50 ng/ml was added to half of the transduced cells. Five days later, cells were harvested and their cell growth rates were analyzed.

### Cell growth analysis

Cells were plated in 96-well plates at a density of 1 × 10^3^ cells/well. Cells were then treated with vehicle (control) or IFN-α for an additional 48 h. Medium was removed and Cell Counting Kit-8 (CCK-8; Dojindo Laboratories, Kumamoto, Japan) solution was added to each well. The plates were incubated for 30 min at 37 ^o^C, and absorbance at 450 nm was measured using a microplate reader.

### Colony-formation assay

Control A549, A549-CHIP-WT, and A549-CHIP-4KR cells were cultured at a density of 500 cells/plate. After 10 days of culture, cells were treated with vehicle or 4000 U/ml IFN-α for an additional 1 week. Colonies were stained with crystal violet.

### Assessment of tumor growth for CHIP-expressing lung cancer cell line in vivo

Tumors were established in the abdomen of 6-week-old male nude mice by subcutaneously injecting 1 × 10^7^ cells of A549 cells transfected with empty vector (mock) or A549 cell lines stably expressing different variants of CHIP (CHIP-WT or CHIP-4KR). The day of implantation was designated as day 0 and IFN-α was administered intratumorally (20,000 unit/mouse) on days 11, 14, 17 and 20. The tumor volumes were calculated using the major axis and minor axis measurements obtained with caliper and following formula: tumor volume = (minor axis in mm)^2^ × (major axis in mm) × 0.523.

### Histology and immunohistochemistry

Representative tumor sections for each group were stained with H&E and then examined using a light microscope (Thornwood, NY, Carl Zeiss Inc.). Additionally, the tumor tissue sections were incubated at 4 °C overnight with mouse anti-PCNA (DAKO, Glostrup, Denmark) to detect proliferating tumor cell population via immunohistochemistry. The slides were counterstained with Meyer’s hematoxylin (St. Louis, MO, Sigma). The positive spots of PCNA were semiquantitatively assessed using the IHC Profiler Plugin for ImageJ software (National Institutes of Health, Bethesda, MD, USA).

### Statistical analysis

Group means were compared using Student’s *t*-test. *P* < 0.05 was considered statistically significant. Values are reported as the mean ± standard error of the mean (S.E.M.).

## Electronic supplementary material

Supplementary Data
