# Supplementary material for: Covalent ISG15 conjugation to CHIP promotes its ubiquitin E3 ligase activity and inhibits lung cancer cell growth in response to type I interferon
Source: Cell Death Dis. 2018 Jan 24;9(2):97. doi: 10.1038/s41419-017-0138-9 (PMC5833375; doi:10.1038/s41419-017-0138-9)
Supplement: Supplementary file 1 — Supplementary Data [file 41419_2017_138_MOESM1_ESM.pdf]

Supplementary Data

Covalent ISG15 conjugation to CHIP promotes its ubiquitin E3 ligase activity and inhibits lung cancer cell growth in response to type I interferon

Lang Yoo, A-Rum Yoon, Chae-Ok Yun, and Kwang Chul Chung

Content: Supplementary Figures and Legends

1. Supplementary Figure S1

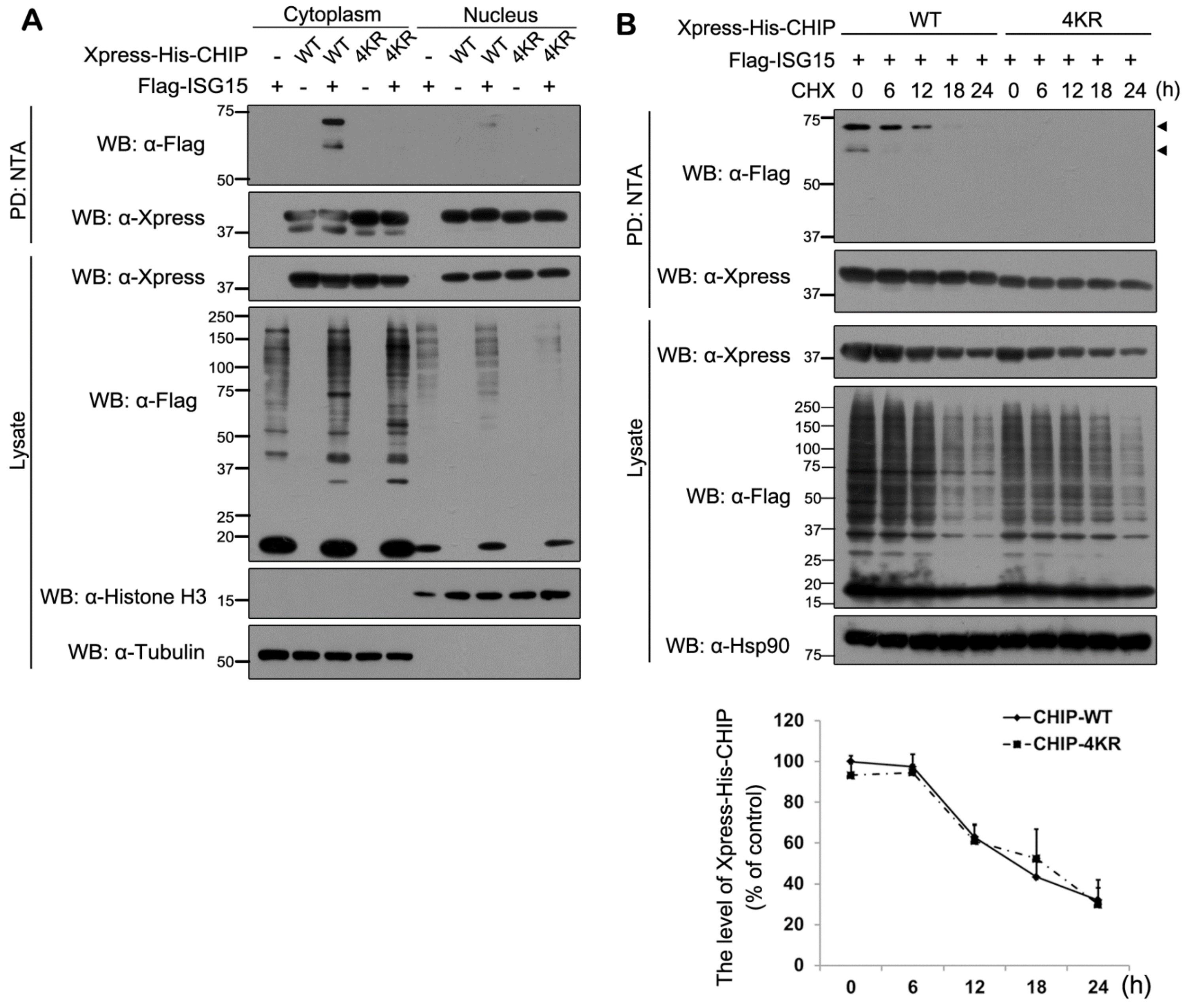

A
Cytoplasm
Nucleus
Xpress-His-CHIP
- WT WT 4KR 4KR - WT WT 4KR 4KR
Flag-ISG15
+ - + - + + - + - +
PD: NTA
WB: α-Flag
WB: α-Xpress
Lysate
WB: α-Xpress
WB: α-Flag
WB: α-Histone H3
WB: α-Tubulin
75
50
37
250
150
100
25
20
15
B
Xpress-His-CHIP
WT
4KR
Flag-ISG15
CHX
0 6 12 18 24 0 6 12 18 24 (h)
PD: NTA
WB: α-Flag
WB: α-Xpress
Lysate
WB: α-Xpress
WB: α-Flag
WB: α-Hsp90
CHIP-WT
CHIP-4KR
The level of Xpress-His-CHIP (% of control)
120
100
80
60
40
20
0
0
6
12
18
24
(h)

Supplementary Figure S1. ISGylation has no effect on CHIP intracellular location or stability. (A and **B)** All samples were transfected with plasmids encoding UBE1L and Myc-UbcH8**. (A)** HEK293 cells were transfected for 24 h with plasmid encoding Xpress-His-CHIP-WT, Xpress-His-CHIP-4KR, or FLAG-ISG15, alone or in combination. Cell lysates were separated into cytosolic and nuclear fraction. Samples were subjected to NTA pull-down (PD: NTA), followed by western blotting (WB) with anti-FLAG or anti-Xpress antibody. Tubulin served as a cytosolic marker and loading control. Histone H3 served as a nuclear marker and loading control. **(B)** HEK293 cells were transfected for 24 h with plasmid encoding Xpress-His-CHIP-WT, Xpress-His-CHIP-4KR, or FLAG-ISG15, alone or in combination and then treated for the indicated times with 20 μg/ml cycloheximide. Cell lysates were subjected to NTA pull-down followed by western blotting with anti-FLAG or anti-Xpress antibody. Hsp90 served as the loading control. Relative Xpress-His-CHIP protein levels at each time point were quantified using MultiGauge version 3.1 program (n = 3; below).
